# Combination therapy of sutimlimab and rituximab can lead to stable remission in autoimmune hemolytic anemia: a case series

**DOI:** 10.1007/s00277-026-07231-6

**Published:** 2026-08-18

**Authors:** Julian List, Anna Katharina Foerster, Alexandra Kutilina, Justus Duyster, Jesus Duque-Afonso, Reinhard Marks

**Affiliations:** Department of Hematology, Oncology and Stem Cell Transplantation, University of Freiburg Medical Center, Freiburg, Germany

**Keywords:** AIHA, Complement inhibition, Sutimlimab, Rituximab

## Abstract

Autoimmune hemolytic anemia (AIHA) is associated with a significant impairment of quality of life for affected patients and can lead to life-threating hemolytic crises. Complement-mediated destruction of antibody-opsonized erythrocytes is a central pathomechanism in many cases of AIHA. Complement inhibition is therefore a promising and logical therapeutic strategy. Sutimlimab, a monoclonal antibody that targets complement factor C1s was approved in 2022 and can effectively inhibit hemolysis. However, this therapy must be administered as a permanent treatment and is only approved for the specific subtype cold agglutinin disease. In this case series, we report on a novel combination therapy of sutimlimab and rituximab, which led to rapid and durable remission in three patients suffering from severe autoimmune hemolytic anemia (two patients with cold agglutinin syndrome and one patient with primary warm AIHA). In two of these patients, treatment with sutimlimab could be discontinued without any recurrence to date. The other patient is also in remission but with ongoing therapy. This highly informative case series reports on an innovative therapeutic concept and may form the basis for further clinical studies in AIHA.

## Introduction

Autoimmune hemolytic anemia (AIHA) is a rare condition leading to an antibody-mediated destruction of erythrocytes. Based on the temperature at which the antibody displays the strongest hemolytic activity, AIHA can be classified as warm, cold, or mixed AIHA [1]. Patients usually present with symptoms of anemia e.g. shortness of breath, paleness, palpitations and general weakness. The severity of the disease can range from mild forms with only slight anemia to life-threatening hemolytic crises. Furthermore, cold antibodies can lead to impaired microcirculation with pain, acrocyanosis or Raynaud-like phenomena especially when patients are exposed to low temperatures [2]. AIHA can occur either as a primary disease or as secondary due to other diseases such as cancers (especially lymphomas), autoimmune disorders and infections as well as to blood transfusions or drugs. In cold-induced AIHA, a further distinction is made between the primary form, cold agglutinin disease (CAD), and the secondary form, cold agglutinin syndrome (CAS). The central pathogenesis in AIHA is the formation of autoantibodies against erythrocytes which can lead to both extravascular and intravascular hemolysis through various mechanisms [3]. Particularly in cold AIHA the complement mediated hemolysis plays a central role in this process [4]. Therapeutic strategies include B-cell depletion, steroids (not effective in cold AIHA), immunosuppressants, chemotherapy, complement inhibitors, splenectomy and, in severe cases and emergencies, plasmapheresis and blood transfusions [5–8]. However, the optimal treatment strategy has not yet been sufficiently explored and AIHA patients have a poorer survival compared to the general population [9]. Therefore, new therapeutic approaches are urgently needed. Here, we describe three patients with severe, life-threatening AIHA who were treated with a combination of rituximab and the complement inhibitor sutimlimab which is the first approved therapy for CAD [10]. We hypothesize that simultaneous B-cell depletion and terminal complement blockade may provide rapid disease control while facilitating sustained immunological remission.

## Case presentations

**Fig. 1:**
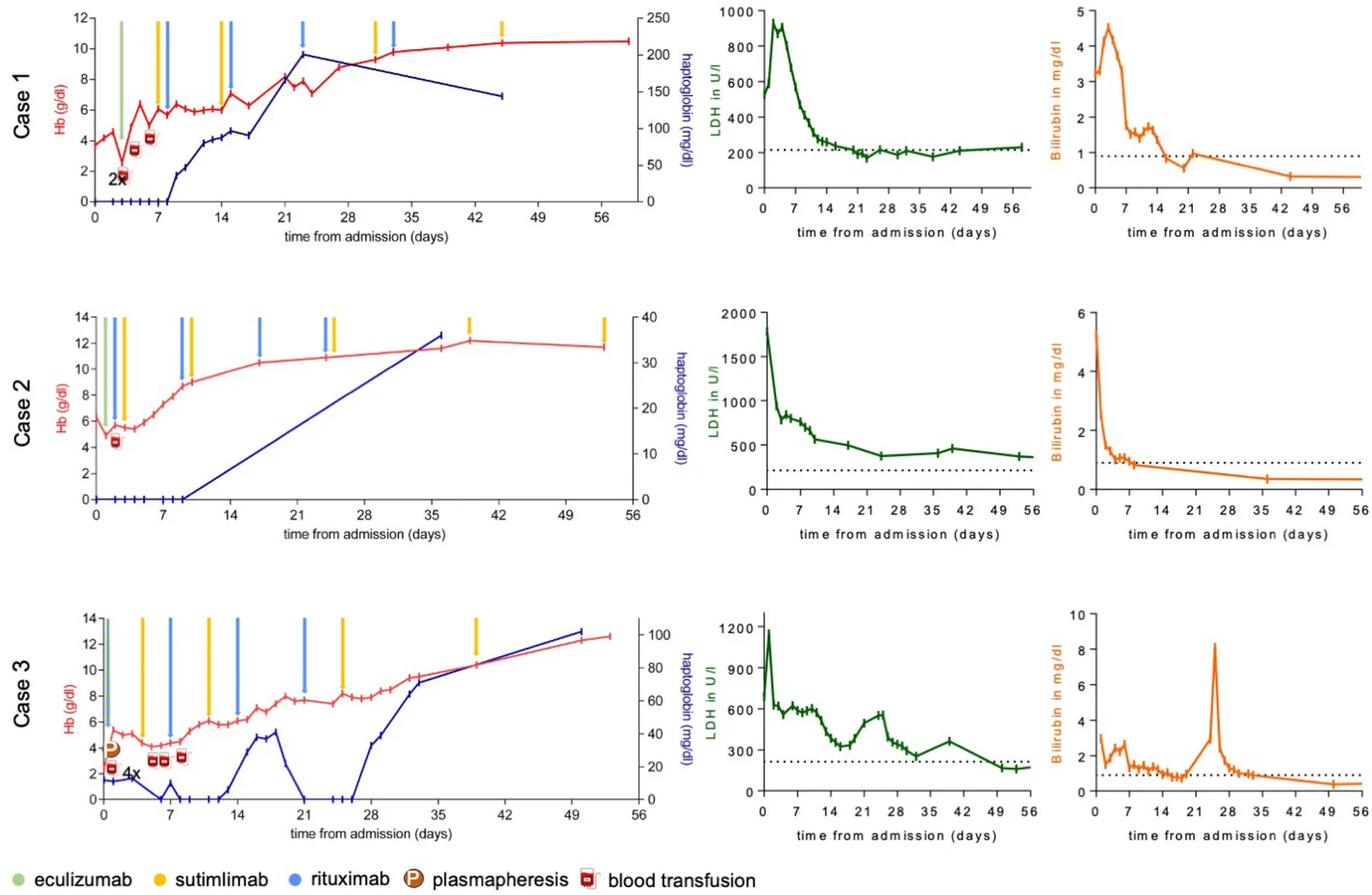
Hemoglobin, haptoglobin, LDH and total bilirubin levels in initial treatment period

All patients were treated with rituximab (four weekly infusions of 375 mg/m^2^). Because the C1s complement inhibitor sutimlimab was not immediately available, bridging complement inhibition with a single dose of eculizumab (900 mg) was initiated in all patients. Upon availability, therapy was switched to weight-adjusted sutimlimab (6500 mg if < 75 kg; 7500 mg if ≥ 75 kg; administered weekly for two doses, then biweekly). Due to the urgency of treatment initiation, vaccination against encapsulated bacteria could not be performed prior to complement inhibition. Given concomitant rituximab therapy and potential impairment of vaccine response, antibiotic prophylaxis with azithromycin was administered. None of the patients presented with cold induced symptoms. The course of hemoglobin and haptoglobin, LDH and total bilirubin levels in relation to the therapies applied for each patient is shown in Fig. 1.

## Case 1

A 54-year-old female was admitted to our hospital in May 2025 with severe AIHA (Hb 3.7 g/dl). The direct antiglobulin test (DAT) was positive and further testing showed cold agglutinins (titer 128). She was previously diagnosed with Hodgkin lymphoma and was treated with eBEACOPP with documented CR in 2023. PET-CT and bone marrow puncture upon admission showed no signs of active lymphoma. The patient was tested positive for rhino/entero-virus in a respiratory panel. Initially, while the antibody differentiation was still pending, the patient received dexamethasone with no response. Due to another drop in Hb levels to 2.6 g/dl accompanied by neurological symptoms, the patient received three units of packed red blood cells which stabilized the patient. At this point we started the complement-inhibiting treatment with eculizumab, followed by rituximab and sutimlimab. With these measures the Hb level could be increased and stabilized around 7 g/dl and the patient could be discharged after 15 days. Prior to the next scheduled outpatient visit the patient was readmitted with grade IV neutropenia and infection, which responded rapidly to G-CSF and antibiotics. The treatment with sutimlimab was continued and the Hb-Levels raised to 11.7 g/dl three months after diagnosis. Further, the levels of haptoglobin, LDH and bilirubin normalized. After six sutimlimab applications the treatment was discontinued due to joint pain and leukopenia/neutropenia in August 2025. At the last follow-up six months after diagnosis the Hb-level was normal at 12.6 g/dl off treatment. The DAT was negative at that timepoint.

## Case 2

A 58-year-old female presented at our emergency department in July 2025 with a presyncope caused by severe hemolysis with an Hb level of 6.3 g/dl, which dropped to 4.9 g/dl the next morning. Remarkably, the patient had been hospitalized for a third relapse of known immune thrombocytopenia (ITP) four weeks earlier and had been successfully treated with steroids and intravenous immunoglobulins (IVIG). Notably, the patient had normal Hb levels during this episode. The ITP was first diagnosed in 1998 during pregnancy and treated with steroids, IVIG and then splenectomy. Therefore, this case could also be classified as Evans syndrome. The DAT was positive. Further testing showed the presence of free and bound warm- and cold-reactive antibodies. The warm- and cold-reactive antibodies were partly attributable to the intravenous immunoglobulin given in the weeks prior due to ITP and laboratory differentiation between transfused antibodies and autoantibodies was not possible. As a result, it is difficult to assign this case to a specific subtype of hemolytic anemia. However, based on the low cold agglutinin titer and the fact that in further testing after 5 weeks DAT was only positive for IgG, we consider this case most likely to be warm AIHA with possible complement activation.

The patient initially received steroids, one unit of packed red blood cells and was also rapidly started on eculizumab. The next day rituximab was added, and the complement inhibition was later switched to sutimlimab. On day 10 after diagnosis the Hb level increased to 9 g/dl and the patient was discharged. Sutimlimab treatment was initially continued in our outpatient clinic but was discontinued after six doses due to the patient’s preference. At the latest follow-up seven months after diagnosis, the patient had an Hb of 14.5 g/dl off treatment. Notably, platelet levels also increased under sutimlimab treatment and remain above the upper limit of normal to date (platelets 579 G/µl at latest follow-up). Haptoglobin and bilirubin levels returned to normal, while LDH levels decreased, but remain slightly elevated. The DAT was negative at the latest follow-up appointment as well.

## Case 3

A 23-year-old male was transferred in October 2025 to our ICU from another hospital with severe cold AIHA and an Hb level of 2.4 g/dl with acute clinical onset after a respiratory infection a few days prior. The patient showed reduced vigilance and was hypotensive requiring vasopressors. The patient was immediately started on steroids, plasmapheresis, IVIG, eculizumab and rituximab and received four units of packed red blood cells. These measures stabilized the patient allowing him to be transferred to our regular hematology ward. We continued treatment administering sutimlimab and rituximab as described above. Immunofixation from serum was slightly positive for IgG kappa. CT scans and a bone marrow puncture did not show an underlying hematological condition. Initially, the patient still required additional blood transfusions. In the further course, the patient’s Hb levels continued to rise, and he was discharged with an Hb level of 7.2 g/dl three weeks after admission and was no longer transfusion-dependent. The patient was readmitted to our ward a few days later with upper abdominal pain and elevated liver enzymes (GPT 769 U/L, γGT 911U/l) as well as elevated bilirubin (total bilirubin 8.1 mg/dl, conjugated (direct) bilirubin 7.22 mg/dl) and suppressed haptoglobin but with a stable hemoglobin of 8.2 g/dl. There was no evidence for infectious hepatitis. Liver ultrasound showed a steatosis hepatis with sludge in the gall bladder but no clear sign of obstruction. Although the haptoglobin was suppressed we did not consider the hemolysis as the primary cause for this event because the patient had a stable hemoglobin. No comprehensive explanation for this episode was found; the laboratory cholestasis constellation may have been an expression of a gallstone passing as clinical symptoms and laboratory markers quickly resolved without additional therapeutic measures. A causal relationship to sutimlimab or rituximab could not be established. The patient was discharged with normal bilirubin and almost normalized liver enzymes. We continued the sutimlimab treatment in our outpatient clinic and the patient is still under sutimlimab treatment (eight applications to date) with an Hb of 14.0 g/l at the last follow-up three months after diagnosis. Haptoglobin, LDH and bilirubin as well as liver enzymes have normalized; the latest DAT was negative.

## Discussion

Combining sutimlimab and rituximab addresses underlying immunological principals of AIHA. The destruction of erythrocytes repeatedly presents new autoantigens to the immune system, which can lead to renewed antibody formation. By rapidly suppressing complement-mediated hemolysis while depleting autoreactive B cells, this approach may interrupt the cycle of antigen exposure and autoantibody production. We speculate that this may lead to better and sustained remissions. Notably, in two of three patients, sutimlimab could be discontinued without relapse (to date) during follow-up, suggesting that combination therapy with rituximab may allow time-limited complement inhibition in selected cases.

An important point of discussion is whether this therapeutic concept can be applied to all types of autoimmune hemolysis, as the influence of the complement system can vary depending on the type (warm vs. cold, primary vs. secondary) [4]. In case 1 and case 3 immunohematological testing revealed cold AIHA (see Table 1) and both patients had a history of respiratory infection before the onset of hemolysis which might indicate a CAS. In case 2, no trigger for AIHA could be identified, indicating primary AIHA in the context of Evans syndrome. The interpretation of the immunohematological testing was challenging due to the previous IVIG treatment but overall, it was considered as a warm AIHA with a possible complement involvement.

**Table 1:** Baseline patient characteristics, hemolysis parameter and immunohematological test results

Case number	1	2	3
sex	female	female	male
age (years)	54	58	23
hemoglobin at admission (g/dl)	3.7	6.3	2.4
lowest hemoglobin measured (g/dl)	2.6	4.9	2.4
haptoglobin at admission (g/dl)	< 5	< 5	12
LDH at admission (IU/l)	525	1783	691
total bilirubin at admission (mg/dl)	3.23	5.23	2.97
AIHA subtype	CAS	primary warm	CAS
monospecific coombs test			
IgA	*-*	*+*	*-*
IgM	*-*	*-*	*-*
IgG	-	+	+
C3d	+	+	+
cold agglutinin titer	1:128	low	1:32–1:64
thermal amplitude	37 °C	37 °C	37 °C
screening after DTT treatment of the sample	N/A	N/A	negative

However, classification of hemolysis in everyday clinical practice is not always unambiguously as real-world AIHA phenotypes often overlap. We speculate that the decisive factor in the effectiveness of a complement-based therapy lies in the detection of complement-deposition on the erythrocytes shown by the coombs test, which was the case in all our patients. Complement deposition on erythrocytes, as demonstrated by DAT positivity for C3d, may thus represent a clinically meaningful biomarker for response to complement-directed therapy, potentially more relevant than classical warm/cold classification alone.

Regarding safety, it should certainly be noted that the patients are at risk of infection because immunosuppression is induced via two different mechanisms. The effect of important vaccinations against encapsulated pathogens, which should ideally be administered prior to treatment with a complement inhibitor, can be weakened by rituximab therapy. We therefore decided to administer antibiotic prophylaxis using azithromycin. We also observed one case of severe neutropenia, which responded well to G-CSF stimulation, but persisting cytopenia with joint pain was ultimately the cause for therapy discontinuation. Whether this might have been a direct side effect of the therapy remains unclear, and more experience is needed. In summary, combination therapy with sutimlimab and rituximab in AIHA is a promising therapeutic approach, but the efficacy and safety must certainly be evaluated in further clinical trials.

## Data Availability

For original data, please contact Julian.List@uniklinik-freiburg.de.
